# Assessing trace elements in soils and rice: insights from the Baixo Vouga Lagunar (Portugal)

**DOI:** 10.1007/s10653-025-02408-w

**Published:** 2025-03-03

**Authors:** Mariana Santos Gama, Luís Portela, Carla Patinha, Nuno Durães

**Affiliations:** GEOBIOTEC & Departamento de Geociências da Universidade de Aveiro, Campus de Santiago, 3810-193 Aveiro, Portugal

**Keywords:** Soil-rice plant system, Potential toxic element, Sequential selective chemical extraction, Bioavailability, Floodwater

## Abstract

Paddy soils undergo wet-dry cycles that greatly influence the behaviour and availability of nutrients, but also of potentially toxic elements (PTEs). This study assessed the quality of paddy soils (actively cultivated and abandoned) and rice (white, brown, and wild) produced in the Baixo Vouga Lagunar (BVL) region, central-north Portugal. Surface soils were analysed for physicochemical parameters and chemical compositions, alongside sequential selective chemical extraction to evaluate metal(loid) availability. Chemical analyses were also performed on interstitial- and irrigation waters, and rice grains. The BVL soils are very strongly to moderately acidic (pH = 4.4–5.8), with organic matter contents reaching up to 34%, and exhibit a wide range of electrical conductivity values. Abandoned rice fields generally show higher values of these parameters and evidence of saline water intrusion. Several sites showed As, Cu, Pb, and U concentrations exceeding Portuguese thresholds for agricultural soils. While Cu levels were similar in both cultivated and abandoned fields, the latter had higher contents of As, Pb, and U. A geogenic origin is envisaged for these metal(loid)s, though anthropogenic contributions cannot be excluded. Sequential selective chemical extraction showed that Pb and U are strongly associated with available fractions, whereas amorphous Fe-oxyhydroxides primarily support As and Cu. Nevertheless, porewaters and irrigation waters showed low concentrations of these PTEs, suggesting minimal mobilisation to water. Furthermore, translocation to rice grains was low, with concentrations well below European Commission limits, indicating that elevated PTEs in soils do not necessarily lead to toxic levels in rice, providing reassurance regarding food safety.

**Supplementary Information:**

The online version contains supplementary material available at 10.1007/s10653-025-02408-w.

## Introduction

Rice is one of the most grown and consumed cereals worldwide, a staple food, and an important source of energy for more than half of the population of the world. Unlike other crops, rice is mostly cultivated under continuous or intermittent flooding conditions, which promote the alternation of oxidising and reducing conditions in the soil. This particularity influences soil dynamics in the emission of greenhouse gases (*e.g.*, Fusi et al., 2014; Gaihre et al., 2014; Miranda et al., 2015), the preservation versus oxidation of organic matter, and the behaviour and availability of nutrients, as well as of metal(loid)s (Figueiredo et al., 2013). The latter, when exceeding the optimal concentrations for the performance of physiological or metabolic functions in plants, or in the case of elements that do not represent any role in the indicated functions, are known as potentially toxic elements (PTEs).

When the availability of these PTEs is favoured in soils, their migration to waters is facilitated, but also their uptake by plants. If these metal(loid)s undergo translocation and accumulation in the aerial parts of plants, namely in the edible parts, this could pose a risk to human health through their ingestion (Kibria et al., 2016). Numerous studies have investigated the accumulation of metal(loid)s in the soil-rice plant system (*e.g.*, Gupta & Gupta, 1998; Fu et al., 2008; Zhu et al., 2008). In certain regions of Asia, high PTE contents have been determined in rice, *e.g.*, elevated As concentrations detected in rice produced in Bangladesh (Abedin et al., 2002; Das et al., 2004; Meharg & Rahman, 2003). According to Williams et al. (2007), the anaerobic/anoxic conditions prevailing in paddy soils, where mobile arsenite (As^III^) predominates, facilitate arsenic uptake by roots and its subsequent transfer to shoots and grains. These authors found that rice tends to absorb arsenic more efficiently than other cereals grown under more oxygenated conditions (*e.g.*, wheat and barley; Williams et al., 2007). Elevated concentrations of Cd, Pb, and As have been reported in rice grains from both contaminated and uncontaminated regions (Meharg et al., 2009, 2013; Mu et al., 2019; Watanabe et al., 1996).

Currently, the Baixo Vouga Lagunar (BVL) is the only region in northern Portugal where rice is produced, although its cultivation has been progressively abandoned due to socioeconomic changes and salinisation of soils. The rice fields of BVL are located in an area of great natural and human wealth, characterised by a unique landscape—a rural intricate system composed of pastureland, rice fields, and wetland areas (marshes and reed beds) and the "Bocage". The Bocage is a structure of fields divided by hedges and trees that limit the cultivated areas, pastures and water lines. It is recognised as an ingenious agro-ecosystem with great biological potential. The rice fields are of great economic and landscape value, representing an important means of subsistence for some farmers. Despite the ecological and economic significance of this region, there remains a notable gap in studies addressing the quality of paddy soils and the broader environmental impacts of rice cultivation in the BVL, stressing the need for further research in this area.

Therefore, the main objectives of this study were to assess: (i) the potential impact of current rice cultivation practices on the quality of BVL soils; (ii) the origin of PTEs in the soils of BVL rice fields (whether geogenic or anthropogenic); and (iii) the influence of soil and floodwaters characteristics on the concentrations of PTEs in the rice produced in the region. For this purpose: (a) paddy soils were characterised based on physical, physicochemical, chemical, and mineralogical parameters; (b) the concentration of the main PTEs in the soils, and in the interstitial- and floodwaters were determined; (c) the environmental availability of the main PTEs in soils was investigated; and (d) the chemical analysis of rice grains grown in the studied soils was performed. Based on the work of Gama (2022), particular emphasis was placed on As, Cu, Pb, and U, as these PTEs exceed the guidelines for agricultural soils established by the Portuguese Environment Agency (APA, 2022). Due to their potential toxicity, persistence in the environment, and risk of accumulation in the food chain (*e.g.*, Zhao et al., 2012; Xiao et al., 2019), studying the behaviour of these elements in flooded soils used for agricultural practices is critical for assessing both environmental and food safety risks.

## Study area

### Geological setting

**Fig. 1:**
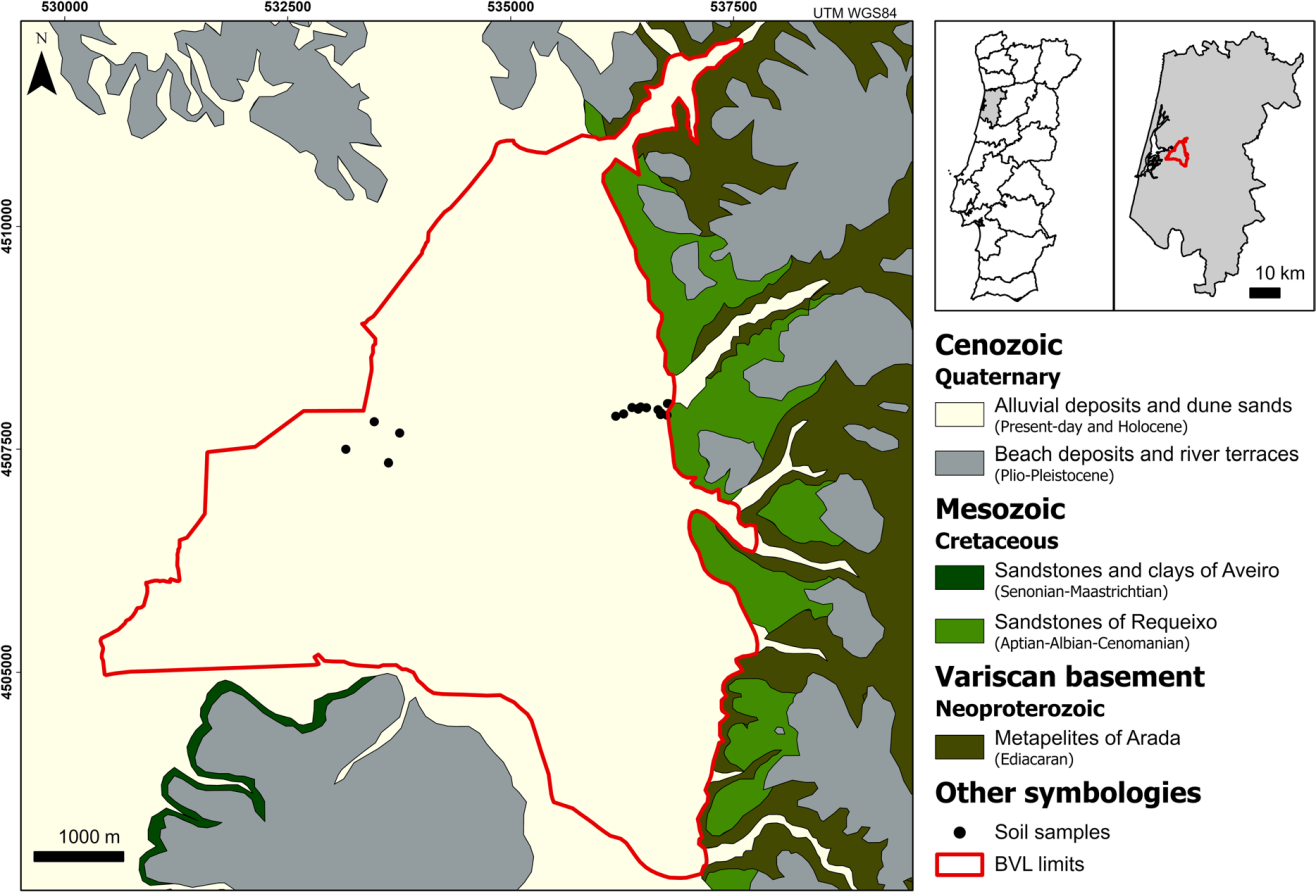
Geological sketch map of the Baixo Vouga Lagunar (BVL), modified from sheets 16-A (Aveiro) (Teixeira & Zbyszewski, 1976) and 13-C (Ovar) (Teixeira, 1963) of the Geological Map of Portugal, scale 1:50 000. The insets on the right show the location of the study area in mainland Portugal and in the Aveiro district

The Baixo Vouga Lagunar is located in the central-north coastal area of mainland Portugal, in the district of Aveiro (Fig. 1). This area is a complex lagoon system formed by fresh- and brackish water environments, resulting from the interaction of the Vouga River and other smaller rivers (freshwater) with the Ria de Aveiro (saltwater).

Referring to geological context, the BVL region is located on the northern extreme of the Western Meso-Cenozoic fringe, an elongated, NNW-SSE-trending morphostructural unit, located along the Atlantic margin of mainland Portugal. This unit essentially comprises sediments of the Lusitanian Basin, which developed during the Mesozoic as a result of the opening up of the North Atlantic. From the Upper Aptian onwards, this western margin was set in a passive margin geodynamic environment, with a predominance of continental or lacustrine sedimentation (Kullberg et al., 2006). The BVL area is dominated by Quaternary sedimentary formations (alluvial deposits, beach sands, and dune sands; Fig. 1), deposited on a Neoproterozoic metamorphic substratum (“Metapelites of Arada”). The latter bound the BVL to the east (Fig. 1) and are essentially composed of dark, low-grade, highly altered metapelites (Teixeira & Zbyszewski, 1976). These rocks are overlain by detrital deposits of Triassic, Cretaceous, and Cenozoic age. The Triassic deposits, not represented in Fig. 1, consist of a basal conglomerate unit that is overlain by banks of poorly calibrated sandstones that gradually transition to pelitic levels (Teixeira & Zbyszewski, 1976). Above this unit lie the Cretaceous sequences, essentially composed of sandstones, clays, and minor limestones. The “Sandstones of Requeixo”, which in the region constitute the base of the Cretaceous, include sandstones of variable grain size with a kaolinitic clay matrix, and with an overall fining-upwards tendency (Teixeira & Zbyszewski, 1976). To the south of the BVL crops out a fluvio-marine series, known as “Sandstones and Clays of Aveiro”, constituted by an alternation of marly sandstones and clays (Teixeira & Zbyszewski, 1976). Quaternary deposits occupy extensive areas in the BVL region and comprise two subsets of detrital nature (Fig. 1; Almeida et al., 2000): (i) fluvial terraces and old beach deposits of Plio-Pleistocene age; and (ii) Holocene to present-day alluvium, beach sands and aeolian sands, which constitute the entire dune cord bordering the Aveiro lagoon. As illustrated in Fig. 1, the BVL region is located in an area essentially composed of alluvial deposits.

### Rice cultivation in the Baixo Vouga Lagunar region

Rice cultivation in the BVL went through different evolutionary processes. In the past, the lack of specific organic herbicides for these crops led to the use of more artisanal cultivation techniques. Thus, rice germination began in nurseries, and only later plants were transferred to the fields. Rice paddies were then manually weeded. The evolution of rice cultivation practices was marked by the mechanisation of agricultural work and the use of fertilisers and herbicides, the latter allowing direct sowing. After mowing the rice with machines, rice stubbles are now left in the fields, contributing to the increase of organic matter in these soils.

Over the past few decades, progressive saline intrusion, combined with socioeconomic changes, has led to abandonment of many rice fields. In the absence of direct anthropogenic intervention, these fields are left uncultivated and unreworked, resulting in dense vegetation and, consequently, high levels of organic matter in soils. Without the typical practice of waterlogging, these lowland fields are mostly dry or contain only a thin layer of water. However, during periods of heavy rainfall, they become saturated or submerged as the phreatic level rises.

## Materials and methods

### Sampling

**Fig. 2:**
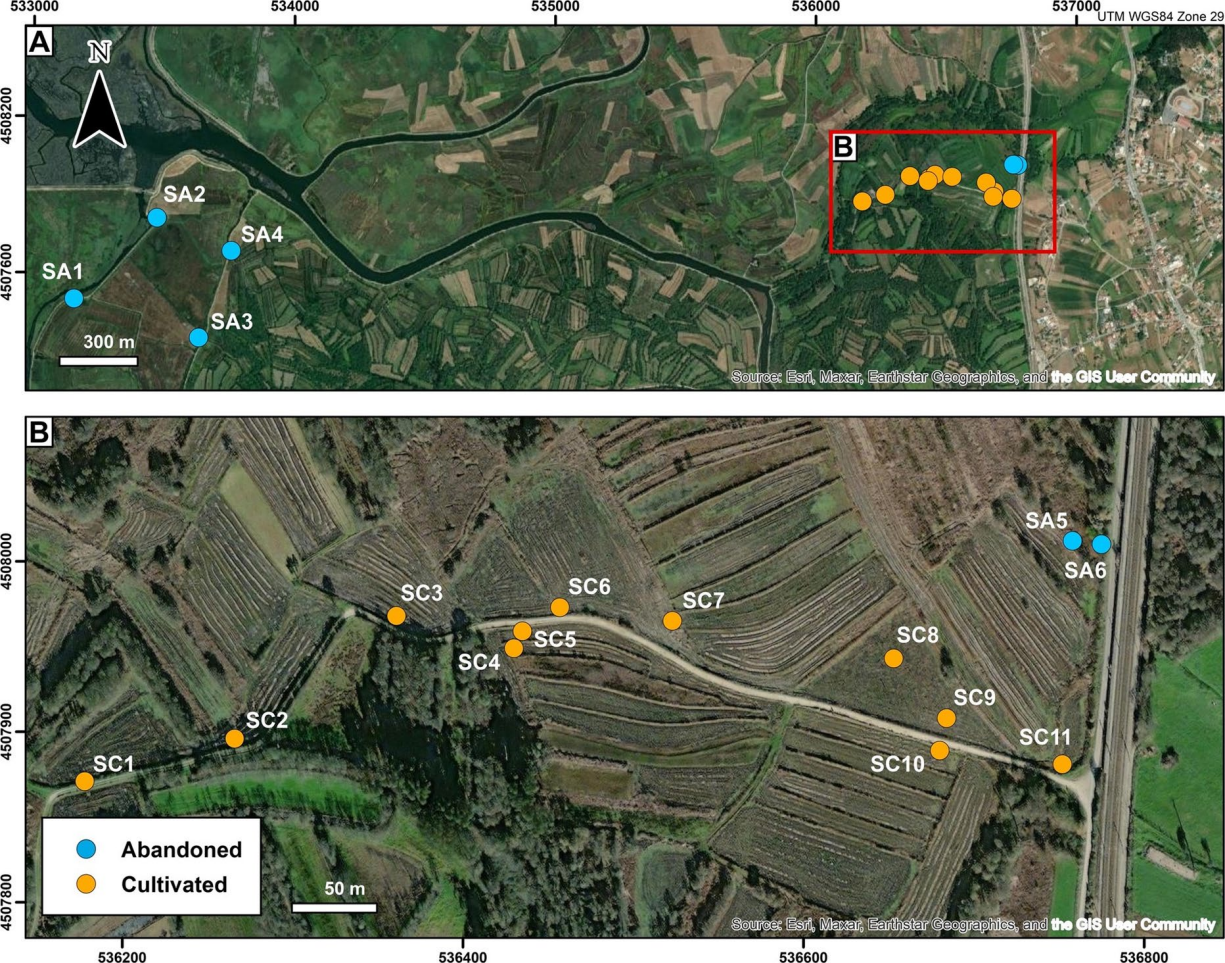
**(A)** Location of the soil sampling sites and **(B)** detail of the sampling area delimited by the red rectangle in image A

Soil and floodwater sampling in the rice fields of the Canelas region (BVL; Fig. 2) involved three separate campaigns, carried out in April and July of 2021 and in March of 2022. A total of 33 soil samples were collected over 17 sampling points, distributed in rice fields abandoned for more than 40 years (SA1-SA6) and actively cultivated paddies (SC1-SC11). Given the close spatial proximity, the influence of the same water column, and the similar physicochemical and geochemical features of samples SA5 and SA6 with soils collected on cultivated fields (Fig. 2), these samples will be treated hereon as cultivated soils. Thirteen floodwater samples were collected at the same sampling sites as soils (adjacent agricultural fields were only sampled once).

Surface soil samples (0–20 cm deep) were collected using a stainless-steel spade and stored in plastic bags, while water samples were collected in polyethylene bottles and kept in a cooler. In the second campaign, before drying and preparing the soil samples of cultivated rice fields, interstitial waters were extracted in the laboratory using standard Rhizon samplers (Rizosphere Research Products). The Rhizon samplers were connected to pre-evacuated 10 mL syringes to collect the pore water with a gentle vacuum.

For this study, five samples of rice harvested from the Canelas paddy fields (studied region) were analysed for their chemical compositions: (a) white rice (polished; short-grain; “Carolino”) produced in 2020, 2021 and 2022; (b) wild rice (husked; long-grain) from the 2022 harvest; and (c) brown rice (husked; short-grain) also produced in 2022.

### Sampling processing

In the laboratories of the Department of Geosciences of the University of Aveiro (DGeo-UA), soil samples were dried in a ventilated oven at *ca*. 40 °C for a minimum of 48 h. After drying, samples were disaggregated in a porcelain mortar and then sieved to a particle size of less than 2 mm. Following this step, about 120 g were obtained by quartering and were subsequently grounded in an agate ring mill for mineralogical and chemical analyses. The remaining portion (not milled) was reserved for determination of physicochemical parameters and sequential selective chemical extraction.

Water samples destined for chemical analysis of cations were filtered through 0.45 µm Millipore nitrocellulose filters using a vacuum unit system, then stored in 50 mL falcon tubes and acidified with 0.5 mL of ultrapure HNO_3_ [1% (v/v)]. All samples were refrigerated (~ 4 °C) until their respective analysis.

Aliquots of rice grain samples were milled to fine powders in an agate ball mill.

### Analytical techniques

#### Determination of soil texture and mineralogy

Soil texture was determined on the fine-earth fraction (< 2 mm) using the weight percentages of sand, silt, and clay separates (Schoeneberger et al., 2012). Fraction separation was carried out via wet sieving using a vertically assembled mesh series with openings of 2 mm, 1 mm, 500 µm, 355 µm, 125 µm, 90 µm, and 63 µm. Granulometric fractions finer than 63 µm (63 to 0.10 µm) were quantified by X-ray monitored sedimentation using a Micromeritics Sedigraph III Plus, applying Stokes’ sedimentation principles and Beer's law of extinction. Sodium metaphosphate (0.1% w/w; *d* = 0.9941 g cm^−3^) was added to 5.8 g of the sample as a dispersing agent, and previously determined sample densities ranging from 2.31 to 2.46 g cm^−3^ were assumed for the analysis.

The mineralogical composition of soils (< 2 mm) was obtained by X-ray diffraction (XRD) on a PANalytical X'Pert Pro diffractometer installed at DGeo-UA. Instrumental conditions were the following: radiation with CuKα anticathode λ = 1.5405 Å, 40 kV, 40 mA, 10 mm divergence gap in automatic mode. The diffractograms were obtained over the 2θ° range from 4° to 70°, through a counting step of 0.0167° per second.

#### Physicochemical parameters of soils

Determination of pH in soil samples was performed in a suspension of soil in distilled water and CaCl_2_ solution (0.01 M), in a 1M_soil_:5V_solution_ ratio (ISO 10390:^2005^). Measurements were made after 1-h stirring followed by 1-h resting period, with a pH meter (model HI 9025; HANNA Instruments®), previously calibrated with two standard solutions (4.01 and 7.01 at 25 ºC). The electrical conductivity (EC) of soils was determined in a 1M_soil_:2V_solution_ suspension of soil in distilled water (Pansu & Gautheyrou, 2006). Measurements were made after 2 h of stirring using a conductivity meter (model 1481–50; Cole-Parmer), previously calibrated with a 1413 μS cm^−1^ (at 25 ºC) standard solution. Determination of organic matter (OM) contents in soils was carried out by loss on ignition, with 7 to 8 g of sample dried at 105 ºC for 24 h, followed by 16 h at 430 ºC (Schumacher, 2002).

Data quality was evaluated by analysing duplicates of 18% of the sample set. Relative standard deviation (RSD) ranged from 0.3 to 9.3% for $\text{pH}_{\text{H}_{2}{\text{O}}}$, 2.6 to 11.1% for $\text{pH}_{\text{Cacl}_{2}}$, 0.8 to 5.8% for EC, and 0.2 to 6.8% for OM.

#### Chemical analyses of soils, waters, and rice

Chemical analysis of soils, waters, and rice was carried out at DGeo-UA by inductively coupled plasma mass spectrometry (ICP-MS—Agilent Technologies—7700Series).

Determination of major and trace element concentrations of soils was performed after acid digestion of the samples (0.25 g) with an inverted aqua regia solution (1 mL HCl + 3 mL HNO_3_). On the other hand, rice grains (0.25 g) were digested in 3 mL HNO_3_ + 2 mL H_2_O_2_ prior to chemical analysis. At least two aliquots of each rice sample were digested and analysed by ICP-MS. Cation analysis of interstitial and flood waters was performed on the filtered and acidified water aliquots.

The accuracy and precision of the methods used in the chemical analysis of soils, waters, and rice were evaluated by including analytical blanks, certified reference materials (CRMs) and sample replicates in each analytical batch. Blank values were below the ICP-MS detection limits. Quantification limits are detailed in tables A.1, A.2 and A.3 (Supplementary Material), with detection limits set at one-third of these values.

The CRMs included TILL-1 (Canadian Certified Reference Materials Project, CCRMP) for soils, ERM-CE278k Mussel Tissue and ERM-BB422 Fish Tissue (Environmental Resources Management, ERM) for rice, and CPA Chem (10 mg L^−^^1^; performed on a diluted aliquot) and SPS-SW1 Surface Water Level 1 (Spectrapure Standards) for waters. Recovery rates ranged from 62 to 126% for TILL-1 (average = 92%), 77 to 121% for ERM-CE278k (average = 97%), and 92 to 111% for ERM-BB422 (average = 100%). Recoveries for CPA Chem and SPS-SW1 varied from 77 to 114% (average = 96%).

For soils and waters, replicates correspond to 10% of the sample set, whereas at least two replicates were analysed per rice sample. For soils, repeatability was generally better than 5% and 10% for major and trace elements, respectively. In waters, duplicate variability exceeded 15% in only 17% of measurements, with 5% of analyses near quantification limits showing variability between 20 and 35%. Elemental analysis of rice also showed greater variability near the quantification limits.

#### Sequential selective chemical extraction

Representative soil samples were selected for sequential selective chemical extraction (SSCE), to determine the main metal(loid)-bearing phases in the study area.

The SSCE procedure followed the main steps proposed by Cardoso Fonseca and Martin (1986) and Cardoso Fonseca et al. (1999), with some adaptations introduced by Patinha (2002). It included a series of six extractions, each step using a dissolution reagent selective for each of the phases hosting the metal(loid)s present in the samples. The steps corresponded to the following sequence of reagents and extraction phases: F1—ammonium acetate (1 M NH_4_OAc; pH 4.5), to extract metal(loid)s in exchangeable and/or soluble salts phases; F2—hydroxylamine hydrochloride (0.1 M NH_2_OH HCL; pH 2.0), to extract metal(loid)s bound to Mn oxides; F3—Tamm solution [0.175 M (NH_4_)_2_ C_2_O_4_-0.1 M H_2_C_2_O_4_; pH 3.3] under darkness conditions, to extract metal(loid)s bound to amorphous Fe-oxyhydroxides; F4—hydrogen peroxide (H_2_O_2_ 35%), to remove metal(loid)s bound to organic matter and/or partially to sulphides; F5—Tamm solution [0.175 M (NH_4_)_2_ C_2_O_4_-0.1 M H_2_C_2_O_4_; pH 3.3] under UV radiation, to extract metal(loid)s bound to crystalline Fe-oxyhydroxides; and F6—reverse aqua regia (HNO_3_-HCl), to extract resistant phases, including some silicates (partially).

Concentrations of the metal(loid)s dissolved in each extraction solution were determined by ICP-MS according to the conditions previously described. The recoveries, *i.e.,* the ratio between the sum of the concentrations obtained in the different stages of the extraction and the amounts obtained by digestion with inverted *aqua regia* (1 mL HCl + 3 mL HNO_3_), were within the accepted range for this methodology (*i.e.*, between 80–120%).

### Statistical analysis

The data were statistically analysed using IBM SPSS Statistics (Version 29.0.2.0) software package. Analysis of Two-Way ANOVA was conducted to determine statistically significant differences between samples collected on distinct campaigns. A probability of less than 0.05 (*p* < 0.05) was taken as the significance level.

## Results and discussion

### Soils

The soils of the BVL rice fields exhibit textures ranging from loam to clay loam and are essentially composed of primary detrital minerals (quartz, muscovite, plagioclase, and potassium feldspar) and secondary alteration minerals (kaolinite). Halite was also identified in soils from abandoned rice fields, most probably caused by the invasion of saline waters. These soils have a mineralogical assemblage that reflects the geology of the units present in the study area, *i.e.*, alluvium resulting from the erosion of crystalline formations (metasediments and granites) dissected by water courses that disembogue in this region.

The determined values for the physicochemical parameters and elemental concentrations of the studied soils, waters and rice are given in tables A.1, A.2 and A.3 (Supplementary Material), respectively. On the presentation of results and discussion that follows regarding pH, EC, OM, As, Cu, Pb and U, each sample consists of the average values of the different campaigns. This approach is substantiated by the two-way ANOVA test (Table 1), which emphasises that there are no statistically significant differences (*p* < 0.05) between soils samples in the distinct campaigns, except for EC (abandoned soils—1st-2nd campaigns) and U (cultivated soils—2nd-3rd campaigns). For the first exception (EC of abandoned soils—1st-2nd campaigns), the observed differences likely result from seasonal variations, *i.e.*, dry season (2nd campaign – July) with high EC values versus wet season (1st campaign – April) with lower EC values. On the other hand, there is great spatial homogeneity of U contents of cultivated soils sampled in the different campaigns, with samples collected on the same fields retrieving systematically consistent U contents (Table A.1). Such coherence in the determined values and the variation in the number of analysed samples, 9 for the 2nd campaign and 3 for the 3rd campaign, explains the observed statistically significant differences.

**Table 1:** Results of the two-way ANOVA test for the physicochemical parameters and PTEs of the studied soils. Statistically significant differences highlighted in bold

	Abandoned			Cultivated		
Campaigns	1st-2nd	1st-3rd	2nd-3rd	1st-2nd	1st-3rd	2nd-3rd
$\text{pH}_{\text{H}_{2}{\text{O}}}$	0.373	0.587	0.890	0.507	0.540	0.879
EC	**0.013**	0.452	0.245	0.939	0.907	0.950
OM	0.247	0.885	0.340	0.777	0.919	0.927
As	0.207	0.498	0.776	0.942	0.940	0.980
Cu	0.558	0.804	0.522	0.857	0.645	0.569
Pb	0.118	0.266	0.915	0.670	0.834	0.623
U	0.477	0.222	0.111	0.478	0.088	**0.035**

**Fig. 3:**
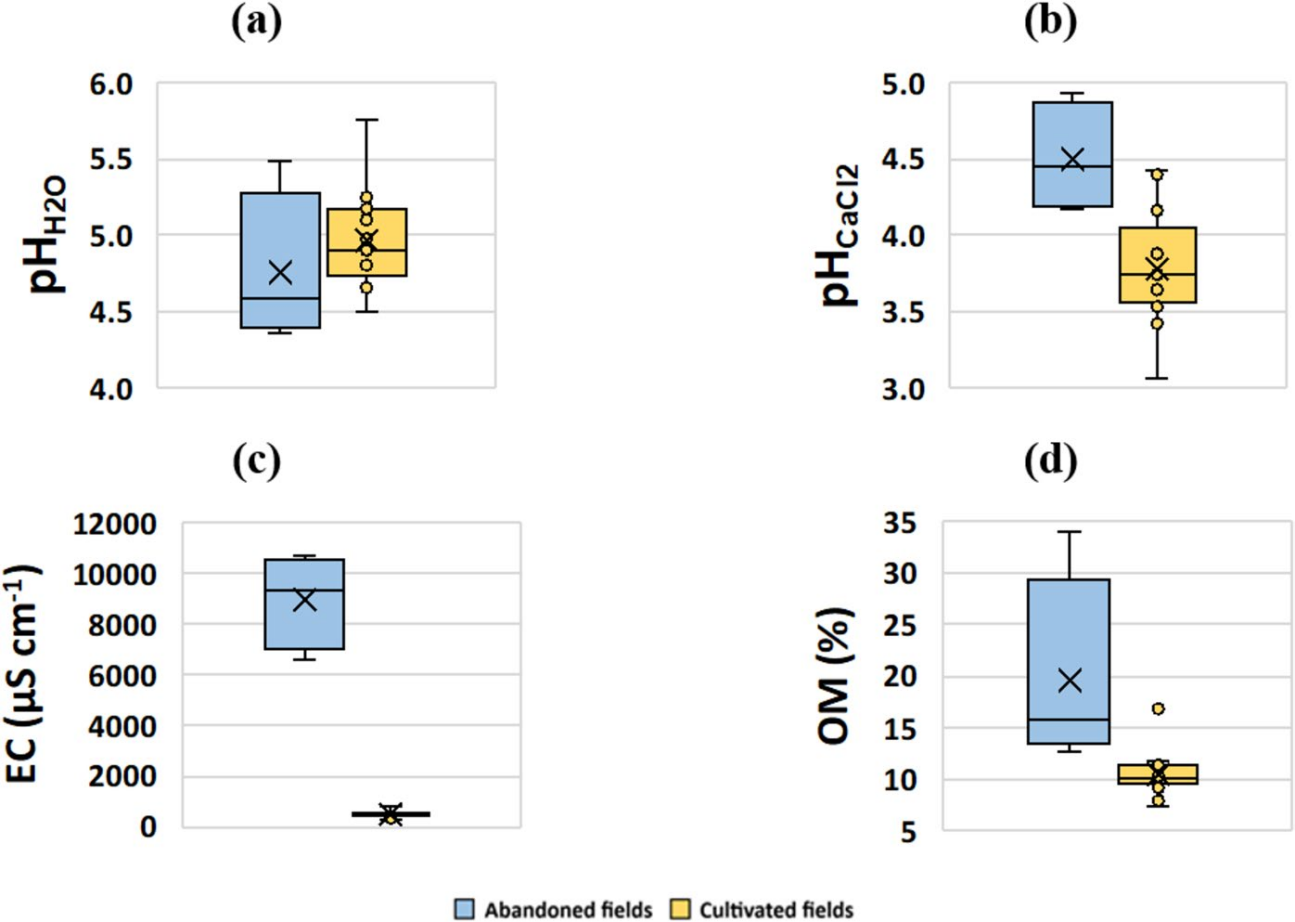
Boxplot diagrams showing the range of values of **(a)** $\text{pH}_{\text{H}_{2}{\text{O}}}$; **(b)** pH CaCl_2_, **(c)** electrical conductivity (EC), and **(d)** organic matter (OM) of the studied soils

Soils of abandoned rice fields exhibit $\text{pH}_{\text{H}_{2}{\text{O}}}$ values between 4.36 to 5.49, whilst those of cultivated paddies vary from 4.50 to 5.76 (Table 2; Fig. 3a). As expected, $\text{pH}_{\text{Cacl}_{2}}$ values are generally lower, with abandoned rice fields displaying values in the range of 4.18 to 4.93, and actively cultivated fields yielding $\text{pH}_{\text{Cacl}_{2}}$ values of 3.06 to 4.42 in (Table 2; Fig. 3b). The $\text{pH}_{\text{H}_{2}{\text{O}}}$ reflects the natural pH of the soil and its interaction with water, while pH CaCl_2_ can provide insights into nutrient availability and soil amendment requirements. The obtained values are similar to those reported by Melo (2018) for soils from other sectors of the BVL region. When using the linear regression model of Libohova et al. (2014) to convert the determined 1M_soil_:5V_solution_ $\text{pH}_{\text{H}_{2}{\text{O}}}$ values to pH 1:1 H_2_O values, the BVL soils can be classified as very strongly acidic to moderately acidic (USDA, 1993). As a rule, low pH values promote the availability of nutrients in solution (McCauley et al., 2009), but also of PTEs, facilitating their uptake by plants and their migration to other soil layers (*e.g.*, Borma et al., 2003).

**Table 2:** Physicochemical parameters of the BVL soils and As, Cu, Pb, and U contents in the studied soils, waters, and rice grains. Electrical conductivity (EC) and soil organic matter (OM) are expressed in µS cm^−1^ and weight%, respectively. Potential toxic element concentrations are given in: soils and rice – mg kg^−1^, pore- and floodwaters – µg L^−1^

	Physicochemical parameters				Potentially toxic elements			
	$\text{pH}_{\text{H}_{2}{\text{O}}}$	$\text{pH}_{\text{Cacl}_{2}}$	EC	OM	As	Cu	Pb	U
*Abandoned paddies*								
*n*	4	4	4	4	4	4	4	4
min	4.36	4.18	6580	12.76	27.36	22.22	48.60	8.24
max	5.49	4.93	10,720	33.91	82.92	84.12	269.2	12.75
median	4.58	4.45	9327	15.85	45.11	34.68	69.15	8.91
mean	4.75	4.50	8988	19.59	50.13	43.87	114.03	9.70
SD	0.50	0.36	1856	9.66	24.25	28.68	104.07	2.07
*Cultivated paddies*								
*n*	13	13	13	13	13	13	13	13
min	4.50	3.06	278	7.48	15.56	26.45	33.21	3.21
max	5.76	4.42	775	16.85	24.13	79.74	60.34	8.29
median	4.90	3.75	494	10.21	21.86	38.81	40.88	5.95
mean	4.97	3.79	506	10.54	20.79	45.08	44.07	5.88
SD	0.33	0.38	138	2.27	2.68	18.34	8.14	1.68
*Porewaters*								
*n*	–	–	–	–	9	9	9	9
min	–	–	–	–	3.01	2.67	0.52	0.24
max	–	–	–	–	19.01	9.57	58.87	3.98
median	–	–	–	–	7.40	4.47	1.01	1.67
mean	–	–	–	–	8.84	5.05	7.63	1.84
SD	–	–	–	–	5.63	2.08	19.24	1.05
*Floodwaters*								
*n*	–	–	–	–	9	9	9	9
min	–	–	–	–	1.10	1.59	0.25	0.05
max	–	–	–	–	7.44	5.01	57.55	0.84
median	–	–	–	–	2.66	2.99	0.55	0.29
mean	–	–	–	–	3.53	3.15	7.14	0.41
SD	–	–	–	–	2.22	1.11	18.92	0.24
*Rice*								
*n*	–	–	–	–	5	5	5	–
min	–	–	–	–	0.073	2.068	< ql	< ql
max	–	–	–	–	0.232	4.913	0.109	< ql
median	–	–	–	–	0.091	2.455	0.035	< ql
mean	–	–	–	–	0.115	2.897	0.050	< ql
SD	–	–	–	–	0.066	1.168	0.041	< ql

^*^n: number of samples; SD: standard deviation; < ql: below quantification limit (< 0.01 mg kg^−1^)

Soils of abandoned rice fields show EC values ranging from 6580 to 10,720 µS cm^−1^, with mean values of 8989 µS cm^−1^ and median of 9327 µS cm^−1^ (Table 2; Fig. 3c). In contrast, cultivated paddy fields show EC values varying from 278 to 775 µS cm^−1^, with mean and median values of 506 and 494 µS cm^−1^ (Table 2; Fig. 3c), respectively. Values of electrical conductivity of a saturated soil paste (ECe) are often used for assessing overall soil salinity levels and their potential impacts on plant growth, while EC 1:2 values can be useful for evaluating the potential impact of salts on plant root zones under more typical field conditions (Corwin & Yemoto, 2017). Expected EC values of a saturation paste extract (ECe) may be estimated using linear regression models from unpublished works using soils from the BVL region. A simple linear regression equation of ECe = 3.9085 × EC_1:2_–0.2665 (R^2^ = 0.9929; values in dS m^−1^) may be used to compute ECe of soils with EC_1:2_ values higher than 2 dS m^−1^. On the other hand, an equation of ECe = 2.3037 EC_1:2_–0.039 (R^2^ = 0.985; values in dS m^−^^1^) is more suited for soils with EC_1:2_ values lower than 2 dS m^−1^. Accordingly, abandoned fields (estimated ECe = 25.45–41.63 dS m^−1^) can be classified as strongly saline, whilst cultivated fields (estimated ECe = 0.60–1.75 dS m^−^^1^) are classified as non-saline (USDA, 1993). The very high EC values observed in abandoned fields reflect the salinisation processes affecting some areas of BVL, as pointed out in previous works (*e.g.*, Melo, 2018).

In the abandoned rice fields, soil OM varies between 12.76 and 33.91% (mean = 19.59%; median = 15.85%), whilst in cultivated fields values vary between 7.48% and 16.85% (mean = 10.54%; median = 10.21%) (Table 2; Fig. 3d). All soil samples from the BVL rice fields exhibit very high OM contents (INIA, 2000), although these are higher in the abandoned fields. Since the latter are not remobilised: (a) oxidation of OM due to aeration is not promoted; and (b) the consequent increase in overall vegetation cover enhances OM production.

Major element (Al, K, Na, Ca, Fe, and Mg; Table A.1) contents are within the common value ranges for uncontaminated soils (Kabata-Pendias, 2011; Reimann & Caritat, 1998) and below the maximum concentration thresholds established for agricultural soils (MECP, 2011; APA, 2022). The concentrations of these elements reflect the main mineralogical phases present in soils. High levels of Al are due to the relative abundance of aluminosilicates (*e.g.*, feldspars, muscovite, and kaolinite) present in all the studied fields. The main sources of K in these soils are muscovite and K-feldspar. Albite-rich plagioclase is the main mineral source of Na and, to a lesser extent, Ca. Abandoned fields exhibit considerably higher Na contents than their cultivated counterparts, which, in line with the presence of halite in some samples, reflects the intrusion of saline waters from the Ria de Aveiro. On the other hand, Ca abundances are consistently higher in cultivated fields, which may be attributed to the addition of Ca-bearing agricultural correctives. Ferromagnesian minerals (*e.g.*, biotite/chlorite) are important sources of Fe and Mg. Additionally, since water courses providing sediments to the BVL region meander through former mining areas, Fe may also originate from sulphides (*e.g.*, Ferreira da Silva et al., 2009). Although such minerals have not been identified by XRD in the < 2 mm fraction, they may be present in accessory amounts in the samples and/or may have suffered oxidation during transport or in the wet-dry cycling of rice cultivation, giving rise to secondary Fe-oxyhydroxides (frequently amorphous).

**Fig. 4:**
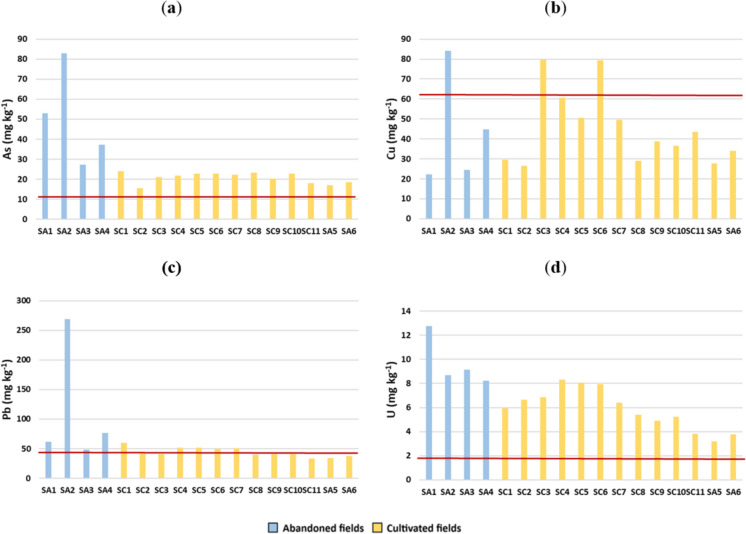
**(a)** Arsenic, **(b)** Cu, **(c)** Pb, and **(d)** U contents in soil samples from BVL rice fields; red lines represent the maximum concentration threshold for agricultural soils according to APA (2022)

In the studied soils, it was possible to detect that some PTEs, namely As, Cu, Pb, and U show high contents (Table 2; Fu et al., 2008; Wong et al., 2002) when considering the reference values of the Portuguese Environment Agency (APA, 2022). The As contents in abandoned fields (27.36–82.92 mg kg^−1^; mean = 50.13 mg kg^−1^; median = 45.11 mg kg^−1^; Table 2; Fig. 4a) are higher than those in cultivated rice fields (15.56–24.13 mg kg^−1^; mean = 20.79 mg kg^−1^; median = 21.86 mg kg^−1^; Table 2; Fig. 4a). Cachada et al. (2019) found that arsenic levels in intertidal surface sediments (0–5 cm) collected in the vicinity of the studied abandoned paddies ranged from 27 to 136 mg kg^−1^. Such contents are much higher than the median values reported by Signes-Pastor et al. (2016) for paddy soils from southern Portugal (15 mg kg^−1^) and Spain (4.2–11 mg kg^−1^). In all the studied samples, arsenic exceeds the national concentration threshold for agricultural soils (11 mg kg^−1^; APA, 2022). Several sources have been proposed for the origin of elevated As contents in paddy soils (Sahoo & Kim, 2013): (1) irrigation with As-rich groundwaters (Meharg & Rahman, 2003; Stroud et al., 2011); (2) mining activities (Kwon et al., 2017); (3) application of pesticides and fertilisers (Quasi et al., 2011; Tang et al., 2020); and (4) natural weathering of rocks and minerals. Based on available data (Condesso de Melo & Marques da Silva, 2008; Melo, 2018), groundwaters of the Aveiro region exhibit low arsenic contents. For this reason, it is believed that the high levels of arsenic in the BVL soils do not originate from the local groundwater. Instead, the remaining three hypotheses are more plausible explanations for the As contents exhibited by the studied soils.

Copper shows similar concentrations in abandoned rice field soils (22.22–84.12 mg kg^−1^; mean = 43.87 mg kg^−1^; median = 34.58 mg kg^−1^) and soils from cultivated rice fields (26.45–79.74 mg kg^−1^; mean = 45.08 mg kg^−1^; median = 38.81 mg kg^−1^; Table 2; Fig. 4b). The determined Cu contents are in perfect agreement with those reported in the survey carried out by Cachada et al. (2019) (25–79 mg kg^−1^). Considering the Cu concentration threshold for agricultural soils provided by APA (2022) (62 mg kg^−1^), such value is only exceeded in three soil samples (Fig. 4b). In addition to natural weathering of soil parent materials, accumulation of copper in agricultural soils has often been reported to result from anthropogenic activities, such as mining and smelting industries, urban, industrial and agricultural wastes, and application of Cu-bearing fertilisers and fungicides (*e.g.*, Cao & Hu, 2000; Hu et al., 2006; Husak, 2015; Tóth et al., 2016; Li et al., 2020). Given the close match between the Cu contents of the studied soils and those of the sediments transported by the rivers that flow into the BVL region (*e.g.*, Nunes, 2007; Ferreira da Silva et al., 2009), it is plausible to admit that sediment inputs play a strong role in controlling the copper budget of the BVL paddy soils.

Regarding Pb contents, values vary between 48.60 and 269.21 mg kg^−1^ in abandoned fields (mean = 114.03 mg kg^−1^; median = 69.16 mg kg^−1^; Table 2; Fig. 4c). In the cultivated fields, Pb concentrations are relatively lower (33.21–60.34 mg kg^−1^; mean = 44.07 mg kg^−1^; median = 40.88 mg kg^−1^; Table 2; Fig. 4c). Available data reveal lead concentrations in the range of 27 to 68 mg kg^−1^ in vicinal areas (Cachada et al., 2019). Similarly to As, Pb concentrations tend to be higher in abandoned rice fields, exceeding in most samples the reference value for agricultural soils (45 mg kg^−1^; APA, 2022). Soil Pb may derive from either geogenic or anthropogenic sources. Amongst the latter, common sources are atmospheric deposition, irrigation, leaded pesticides, and fertilisation (Feng et al., 2019; Huang et al., 2015; Liu et al., 2019). The higher levels of Pb found in the abandoned paddies suggest a geogenic origin for this element. In the cultivated fields, where the soil is periodically remobilised, homogenisation between Pb concentrations of surface and deep layers could have taken place, thus explaining the narrower range of Pb contents of these soils.

In the case of U, higher concentrations tend to be found in abandoned fields (8.24–12.75 mg kg^−1^; mean = 9.70 mg kg^−1^; median = 8.91 mg kg^−1^), compared to actively cultivated rice fields (3.21–8.29 mg kg^−1^; mean = 5.88 mg kg^−1^; median = 5.95 mg kg^−1^; Table 2; Fig. 4d). All samples exhibit U concentrations exceeding those of the reference value for agricultural soils (1.9 mg kg^−1^; APA, 2022). Phosphate fertilisers can be major sources of U in soils, since this element is frequently found as an impurity (Taylor, 2007; Yamaguchi et al., 2009; Kabata Pendias, 2011). On the other hand, a natural origin for this element may be implied, since enrichment of uranium in sediments/soils is expected in reducing or oxygen-poor environments, where U^6+^ is reduced to insoluble U^4+^ (Wignall & Twitchett, 1996).

The results show clear differences in PTE concentrations between abandoned and cultivated sites. The latter exhibit a much narrower range of PTE concentrations compared to abandoned fields, suggesting that soil remobilisation during agricultural activities plays a key role in the redistribution and homogenisation of PTEs in paddy soils. To better assess the origin of these PTEs, whether geogenic or anthropogenic, local observations and available geochemical data may provide valuable insights.

Firstly, information from local communities indicates that rice cultivation practices were largely traditional, with minimal or no pesticide use, although this cannot be completely ruled out. Secondly, geochemical analyses of a sediment core (Teixeira, 2021) drilled a few kilometres west of the abandoned fields revealed As contents exceeding national guideline levels (> 11 mg kg^−1^) in samples up to 3.5 m deep, while Pb and Cu concentrations were generally lower than those found in this study. It is worth noting, however, As, Pb and Cu concentrations increased in the upper layers, with marked peaks in the topmost layer (1.1 m deep), likely pointing to recent anthropogenic inputs. Lastly, whole-rock elemental data from the “Metapelites of Arada” indicate As concentrations (17–18 mg kg^−1^) above the Portuguese threshold (Beetsma, 1995). In the light of this, while the source of these PTEs remains uncertain, the evidence appears to support a dominant geogenic origin, or possibly a mixed contribution from both sources.

**Fig. 5:**
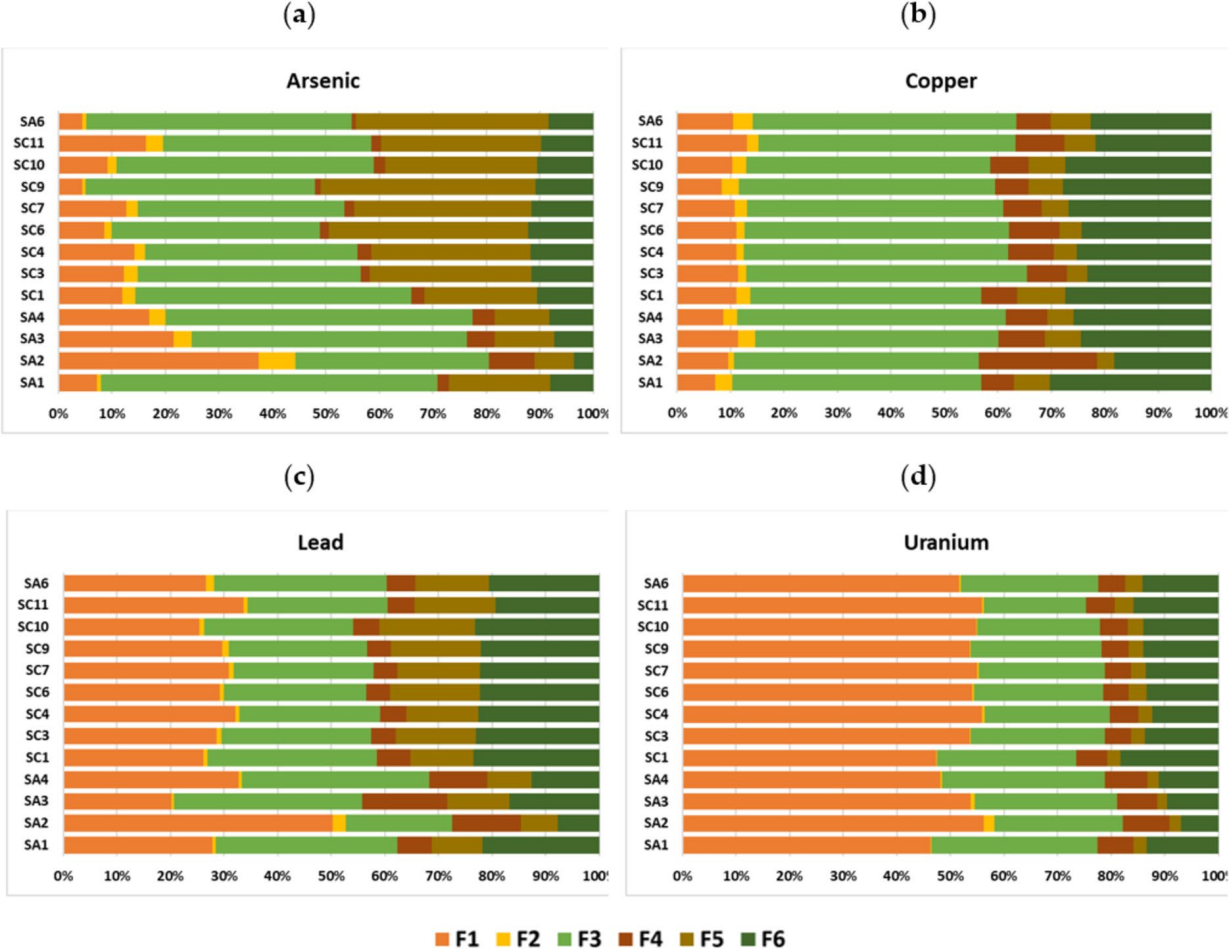
Extraction percentage of **(a)** As, **(b)** Cu, **(c)** Pb and **(d)** U obtained by sequential selective chemical extraction (SSCE) in selected soil samples: (F1) Ammonium acetate–exchangeable phases and/or soluble carbonate phases; (F2) Hydroxylamine Hydrochloride–oxides of Mn; (F3) Tamm in the dark–amorphous Fe-oxyhydroxides; (F4) Hydrogen peroxide–organic matter and sulphides (partially); (F5) Tamm under U.V.–crystalline Fe-oxyhydroxides; (F6) Aqua regia–resistant sulphides or silicates (partially)

Considering the presence of high concentrations of some PTEs in soils, their availability was assessed using the SSCE method. According to the SSCE data for As (Fig. 5a), the most expressive extraction percentage corresponds to the phase of amorphous Fe-oxyhydroxides (F3), both in abandoned (36–63%) and cultivated fields (39–52%; Fig. 5a). In the latter, crystalline Fe-oxyhydroxides (F5) are the second major support phase of this metalloid (21–40%; Fig. 5a). In the abandoned fields (Fig. 5a), only sample SA1 follows this trend (19%), while the remaining samples have the exchangeable and/or soluble fraction as the second most significant extraction phase (17–38%). Strong associations between As and Fe oxides are widely documented in the literature (*e.g.*, Dias et al., 2009). According to Takahashi et al. (2004), during dry periods in rice fields, As is strongly incorporated into Fe-oxyhydroxides. On the other hand, during flooding periods, it is readily released from the soil to water, due to the dissolution (through reduction processes) of Fe-oxyhydroxides and the reduction of pentavalent arsenic (As^V^) to trivalent arsenic (As^III^) (Dixit & Hering, 2003; Smedley & Kinniburgh, 2002).

In both groups of soils, Cu is predominantly extracted in the phase of amorphous Fe-oxyhydroxides (abandoned fields: 46–50%; cultivated fields: 43–53%). As a transition metal, it is expected that Cu has affinity to be incorporated into the structure of oxides (Schwertmann & Taylor, 1989). In fact, the chemical structure of Fe-oxyhydroxides, particularly amorphous ones (with large specific surface and electrical charge deficits), makes them important ion scavengers (Contin et al., 2007). The second most expressive phase of Cu extraction is the residual fraction (abandoned fields: 18–30%; cultivated fields: 22–28%) (Fig. 5b). In the remaining stages, this element is extracted in fairly similar proportions between samples, irrespective of being collected on abandoned or cultivated fields. However, sample SA2 (Fig. 5b) shows a different behaviour, having organic matter and sulphides (22%) as the second most important support phase, followed by the residual fraction (18%).

The highest percentages of Pb extraction are associated with the exchangeable/soluble phases (abandoned fields: 20–50%; cultivated fields: 25–34%), amorphous Fe-oxyhydroxides (abandoned fields: 20–35%; cultivated fields: 26–32%) and the residual phase (abandoned fields: 8–22%; cultivated fields: 19–23%) (Fig. 5c). As with other elements, sample SA2 (Fig. 5c) exhibits a slightly different behaviour compared to the other samples, showing higher percentages of extraction in the following phases: amorphous Fe-oxyhydroxides (20%), organic matter and/or sulphides (13%) and resistant phases (8%). Although Pb has little mobility, it is easily solubilised in environments with acidic pH (*e.g.*, Zeng et al., 2011), which is consistent with the acidic character of the studied soils ($\text{pH}_{\text{H}_{2}{\text{O}}}$ = 4.36–5.76; Fig. 3a), and may explain the high proportion of this element associated with exchangeable/soluble phases.

In the case of U, the highest percentages of extraction correspond to the exchangeable/soluble phases (abandoned fields: 46–56%; cultivated fields: 47–56%), followed by amorphous Fe-oxyhydroxides (abandoned fields: 24–31%; cultivated fields: 19–26%) and residual phases (abandoned fields: 7–13%; cultivated fields: 12–18%), behaving in the same manner for all soil samples and groups (Fig. 5d). Like Pb, the mobility of U is strongly dependent on the Eh–pH conditions of the system (Kabata-Pendias, 2011). The mobility of U can be limited both by the formation of weakly soluble precipitates (*e.g.*, oxides) and by adsorption on clay minerals and organic matter (Kabata-Pendias, 2011). Given uranium is predominantly extracted in phase 1 of SSCE, it is reasonable to assume that it is adsorbed (as an exchange phase) on clay minerals and organic matter, both abundant components in these soils.

### Floodwaters and porewaters

Contents of major and trace elements in floodwater do not show substantial differences between samples collected during different campaigns. For this reason, and for sake of simplicity, average elemental concentrations are presented for samples collected on the same sites (Table A.2).

When only considering the most concerning PTEs in soils (As, Cu, Pb and U), relatively low concentrations were found both in interstitial waters (As: 3.01–19.01 µg L^−1^; Cu: 2.67–9.57 µg L^−1^; Pb: 0.52–58.87 µg L^−1^; U: 0.24–3.98 µg L^−1^) and floodwaters (As: 1.10–7.44 µg L^−1^; Cu: 1.59–5.01 µg L^−1^; Pb: 0.25–57.55 µg L^−1^; U: 0.05–0.84 µg L^−1^) (Table 2). As expected, the former tend to display higher contents of As, Cu, Pb, and U, but, nevertheless, neither the interstitial waters nor the floodwaters show levels that exceed the Portuguese legislation values for irrigation waters (DL 236/98). At first, such low contents of these elements are at odds with the relatively high extraction percentages of the available fraction (F1 phase), particularly for Pb and U (Fig. 5c and 5d). However, such low concentrations in both interstitial waters and floodwaters may reflect the low availability of these metal(loid)s from soils to waters, or even their reprecipitation due to changes in the physicochemical conditions of waters.

### Rice grains

Chemical analyses carried out on the rice grain samples revealed that total-As (t-As) contents vary between 0.07 mg kg^−1^ (brown rice) and 0.23 mg kg^−1^ (2022 wild rice; Table 2; Table A.3). The determined values are within the range of arsenic contents in rice grain from world market (0.11 to 0.28 mg kg^−1^) (*e.g.*, Heitkemper et al., 2001; Williams et al., 2005; Jorhem et al., 2008a; Mondal & Polya, 2008; Meharg et al., 2009; Pinto et al., 2016). According to CR 2023/465  of the European Commission, the maximum admissible content of inorganic-As (i-As) for polished or white rice is 0.15 mg kg^−1^. Following the European Food Safety Authority report (EFSA, 2014), inorganic-arsenic represents *ca*. 70% of total-As. Nevertheless, the total-As contents of the studied white rice samples (0.07–0.10 mg kg^−1^) are consistently lower than the legislated values of 0.15 mg kg^−1^ for inorganic arsenic. In a similar manner, for the remaining types of rice, neither t-As (wild rice: 0.23 mg kg^−1^; brown rice: 0.08 mg kg^−1^) nor the estimated inorganic-As contents (wild rice: 0.16 mg kg^−1^; brown rice: 0.06 mg kg^−1^) exceed the maximum values for husked rice (i-As = 0.25 mg kg^−1^; CR2023/465).

The BVL rice contains Cu contents varying between 2.07 mg kg^−1^ (Carolino rice 2020) and 4.91 mg kg^−1^ (brown rice; Table 2). Copper abundances are slightly lower in polished grains. Comparable results were reported by Lin et al. (2004), Jorhem et al. (2008b), and Pinto et al. (2016).

Lead contents range from below the detection limit (< 0.01 mg kg^−1^; Carolino rice 2022) to 0.11 mg kg^−1^ (Carolino rice 2021) (Table 2), within the range of values reported by other authors (Jorhem et al., 2008b; Shimbo et al., 2001; Zhang et al., 1996). The determined values are substantially lower than the maximum levels presented by the European Commission for cereals (0.20 mg kg^−1^; CR1881/2006).

All samples display uranium contents below the ICP-MS quantification limit (< 0.01 mg kg^−1^; Table 2).

Overall, despite the elevated PTEs contents in soils, their uptake by rice plants and translocation to grains is negligible.

## Conclusions

Paddy soils of the Baixo Vouga Lagunar reveal elevated concentrations of As, Cu, Pb, and U, with some samples exceeding the Portuguese reference values for agricultural soils. The highest levels of As, Pb, and U are generally found in abandoned fields, while Cu levels are similar in both types of fields. Although the origin of these PTEs remains uncertain, the data appear to support a dominant geogenic origin, with a possible mixed contribution from both geogenic and anthropogenic sources. In interstitial and floodwaters, the contents of As, Cu, Pb, and U do not exceed the maximum admissible limits for irrigation water, which reflects the low availability of these metal(loid)s in soils, or their reprecipitation due to physicochemical conditions of these waters.

Although Pb and U are predominantly associated with exchangeable/soluble phases (as shown by the SSCE results), these metals exhibit low concentrations in interstitial and floodwaters, which may be due to the Eh–pH conditions of the waters that do not facilitate their mobility. On the other hand, the extraction of As and Cu, associated with amorphous Fe-oxyhydroxides, is explained by the alternation of reducing (flood) and oxidising (dry) conditions prevailing in this type of culture.

Although the BVL paddy soils are enriched in some PTEs, rice produced in these fields revealed low concentrations of these trace elements. For future work, in order to better assess PTEs (particularly As) accumulation in rice grains, we suggest systematically collecting soil-rice plant pairs along a transect and analysing them for their chemical contents.

## Supplementary Information

Below is the link to the electronic supplementary material.

Supplementary file1 (DOCX 14 KB)

Supplementary file2 (XLSX 23 KB)

Supplementary file3 (XLSX 22 KB)

Supplementary file4 (XLSX 12 KB)

## Data Availability

No datasets were generated or analysed during the current study.
