# Supplementary material for: Assessing trace elements in soils and rice: insights from the Baixo Vouga Lagunar (Portugal)
Source: Environ Geochem Health. 2025 Mar 3;47(4):96. doi: 10.1007/s10653-025-02408-w (PMC11876284; doi:10.1007/s10653-025-02408-w)
Supplement: Supplementary file 1 — Supplementary file1 (DOCX 14 KB) [file 10653_2025_2408_MOESM1_ESM.docx]

**Supplementary Material – Tables captions**

**Table A.1.** Physicochemical parameters and concentrations of major, minor and trace elements of the BVL soils.

**Table A.2.** Concentrations of major anions and major and trace elements of the BVL waters (floodwaters and pore waters).

**Table A.3.** Concentrations of major, minor and trace elements of rice grains.
